# 0854. Metabolomic changes by mass spectrometry in lung tissue from septic rats with mechanical ventilation-induced lung injury

**DOI:** 10.1186/2197-425X-2-S1-P63

**Published:** 2014-09-26

**Authors:** Y Rojas, S Naz, N Nin, A Garcìa, A Ferruelo, L Martínez-Caro, M de Paula, C Barbas, JA Lorente

**Affiliations:** Centro de Investigación Biomédica en Red de Enfermedades Respiratorias (CIBERES), Getafe, Spain; Intensive Care Service, Hospital Universitario de Getafe, Getafe, Spain; Facultad de Farmacia, Centro de Metabolómica y Bioanálisis (CEMBIO), Universidad CEU San Pablo, Madrid, Spain; Intensive Care Service, Hospital Español, Montevideo, Uruguay; Intensive Care Service, Hospital de Torrejon, Madrid, Spain; Hospital Universitario de Getafe, Getafe, Spain; Universidad Europea de Madrid, Madrid, Spain

## Objective

To identify metabolomic changes in lung tissue associated with lung injury induced by mechanical ventilation (VILI) in animals with sepsis, using for the first time a global unbiased metabolomic fingerprinting approach.

## Methods

Rats received cecal-ligation and puncture (CLP) or sham operation, and 24 h later underwent mechanical ventilation for 2.5 h with either V_T_=9 ml/kg, positive end-expiratory pressure (PEEP)=0 cm H_2_O (n=9 and n=12, without and with CLP, respectively); or V_T_=25 ml/kg, PEEP=5 cm H_2_O (n=13 and n=12, without and with CLP, respectively). Lung tissue samples were obtained and analyzed by nontargeted global fingerprinting approach for lung tissue analysis, applying multiple complementary analytical techniques, including liquid cromatography-mass spectrometry (MS), gas cromatography-MS, and capillary electrophoresis-MS. We followed the Principles of Laboratory Animal Care (2010/63/UE 22-09, RD 53/2013 BOE 1-02, ley 32/2007 BOE 7-11).

## Results

Metabolomic changes characteristic of sepsis and VILI were identified. Lung tissue samples from septic rats with VILI were characterized by a specific metabolomic profile as compared to samples from septic rats without VILI. Metabolomic changes indicated increased oxidative stress, and changes in purine, energy, carnitine, aminoacid, urea cycle, vitamines, collagen, ceramide-sphingomyelin and phospholipid metabolism.

## Conclusion

A particular metabolomic profile can be identified in lung tissue from septic rats with lung injury induced by mechanical ventilation.

